# Exploring the best monochromatic energy level in dual energy spectral imaging for coronary stents after percutaneous coronary intervention

**DOI:** 10.1038/s41598-021-97035-7

**Published:** 2021-09-02

**Authors:** Qian Liu, Yajuan Wang, Haicheng Qi, Yaohui Yu, Yan Xing

**Affiliations:** Imaging Center, The First Affiliated Hospital of Xinjiang Medical University, Urumqi, Xinjiang 830011 China

**Keywords:** Cardiology, Cardiovascular diseases

## Abstract

In this study, the optimal monochromatic energy level in dual-energy spectral CT required for imaging coronary stents after percutaneous coronary intervention (PCI) was explored. Thirty-five consecutive patients after PCI were examined using the dual-energy spectral CT imaging mode. The original images were reconstructed at 40–140 keV (10-keV interval) monochromatic levels. The in-stent and out-stent CT values at each monochromatic level were measured to calculate the signal-to-noise ratio(SNR) and contrast-to-noise ratio (CNR) for the vessel and the CT value difference between the in-stent and out-stent lumen (dCT (in–out)), which reflects the artificial CT number increase due to the beam hardening effect caused by the stents. The subjective image quality of the stent and in-stent vessel was evaluated by two radiologists using a 5-point scale. With the increase in energy level, the CT value, SNR, CNR, and dCT (in–out) all decreased. At 80 keV, the mean CT value in-stent reached (345.24 ± 93.43) HU and dCT (in–out) started plateauing. In addition, the subjective image quality of the stents and vessels peaked at 80 keV. The 80 keV monochromatic images are optimal for imaging cardiac patients with stents after PCI, balancing the enhancement and SNR and CNR in the vessels while minimizing the beam hardening artifacts caused by the stents.

## Introduction

Coronary artery disease has ranked high among the leading causes of death and disability in the worldwide, posing a serious threat to the health of the population^1,2^. With continuous improvement of the equipment and optimization of procedures, percutaneous coronary stent intervention (PCI) has become safer and easier to operate than before, and it is regarded as one of the most effective methods to improve myocardial perfusion and alleviate clinical symptoms^3^. However, a major clinical problem is postoperative stent thrombosis, which can lead to in-stent restenosis (ISR), bringing many risks such as potential recurrent cardiovascular adverse events, seriously affecting the long-term prognosis of patients^4^.

Although there has been continuous improvement in CT scanning technology in recent years, overestimation of the CT value in the stent caused by the beam hardening artifacts of the wire harness and the partial volume effect leads to the unclear display of the in-stent lumen and the stent structure itself^5,6^. At present, noninvasive assessment of restenosis after PCI remains challenging^7^. It has been proven that dual-energy spectral CT imaging can reduce the beam hardening effect, which is expected to be an effective method for noninvasive in-stent lumen observation^8–10^. During a dual energy CT scan, data sets both at high and low energy are acquired. These polyenergetic datasets allow for the calculation and reconstruction of virtual monoenergetic images. The underlying energy levels of these reconstructions are reported in kiloelectron-volts (keV). While low-energy images result in higher low-contrast resolution because of a higher attenuation of soft X-rays, high-energy images show less metal artifacts due to the decreased interaction of hard X-rays with dense material^11^. However, the massive data of dual-energy spectral CT imaging increase the difficulty of clinical application, therefore this study aimed to screen the optimal monochromatic energy level for the evaluation of coronary stents after PCI to optimize dual-energy spectral imaging of coronary stents.

## Results

### Subjective assessment

The 80 keV monochromatic level had higher image scores than the other monochromatic levels, and the differences were statistically significant (p < 0.05). Compared with the 40 keV monochromatic level, the maximum image score of the 80 keV image increased from 3.83 to 4.17 (radiologist 1) and 3.86–4.14 (radiologist 2). In contrast, compared with the 140 keV monochromatic level, the maximum image score of the 80 keV image increased from 3.63 to 4.17 (radiologist 1) and 3.60–4.14 (radiologist 2).

### Objective assessment

**Figure 1:**
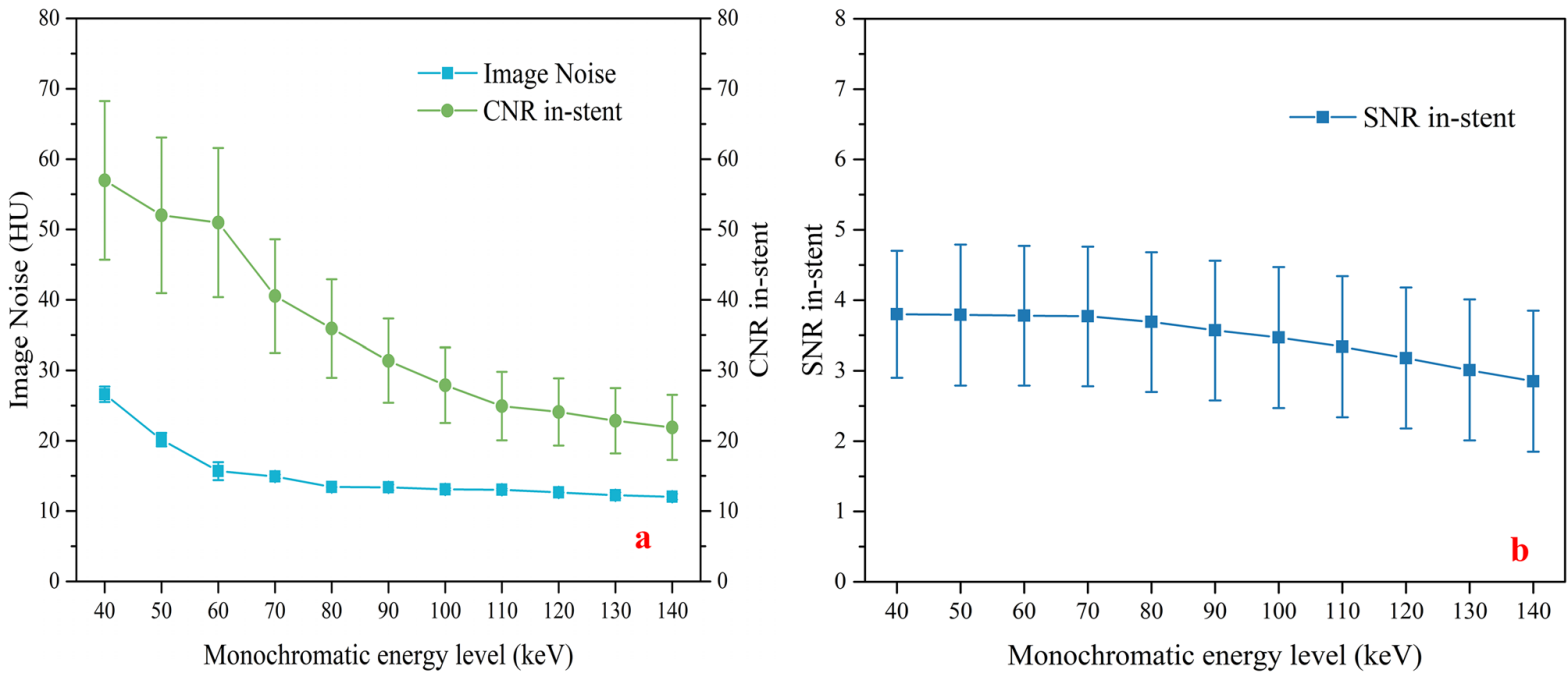
Comparison of image noise and CNR of in-stent (**a**) and comparison of SNR of in-stent (**b**) at monochromatic energy levels between 40 and 140 keV in 10-keV increments.

Image noise, signal-to-noise ratio (SNR) and contrast-to-noise ratio (CNR) of in-stent decreased with the increase in monochromatic energy level: [40–80 keV: Noise: (26.61 ± 1.09)–(13.42 ± 0.01), SNR: (3.80 ± 0.90)–(3.69 ± 0.99), CNR: (56.97 ± 11.27)–(35.92 ± 6.99)]; [90–140 keV: Noise: (13.37 ± 0.40)–(12.04 ± 0.41), SNR: (3.57 ± 0.99)–(2.85 ± 1.00),CNR: (31.37 ± 5.98)–(21.90 ± 4.62)] (Table 1, Fig. 1).

**Table 1:** Mean CT value In-stent and dCT(in–out) as well as image noise values and SNR of in-stent and CNR of in-stent for selected kiloelectron (keV).

	40 keV	50 keV	60 keV	70 keV	80 keV	90 keV	100 keV	110 keV	120 keV	130 keV	140 keV
Noise (HU)	26.61 ± 1.09	20.15 ± 0.96	15.68 ± 1.28	14.91 ± 0.27	13.42 ± 0.01	13.37 ± 0.40	13.08 ± 0.18	13.05 ± 0.14	12.65 ± 0.03	12.25 ± 0.41	12.04 ± 0.41
SNR In-stent	3.80 ± 0.90	3.79 ± 1.00	3.78 ± 0.99	3.77 ± 0.99	3.69 ± 0.99	3.57 ± 0.99	3.47 ± 1.00	3.34 ± 1.00	3.18 ± 1.00	3.01 ± 1.00	2.85 ± 1.00
CNR In-stent	56.97 ± 11.27	52.01 ± 11.05	50.98 ± 10.59	40.54 ± 8.06	35.92 ± 6.99	31.37 ± 5.98	27.89 ± 5.36	24.92 ± 4.86	24.09 ± 4.78	22.84 ± 4.65	21.90 ± 4.62
CT value In-stent (HU)	1242.75 ± 293.72	823.34 ± 217.5	607.10 ± 159.50	449.75 ± 118.52	345.24 ± 93.43	288.44 ± 80.83	242.17 ± 69.70	211.33 ± 63.24	188.71 ± 59.35	170.80 ± 56.71	155.27 ± 54.45
dCT (in–out) (HU)	174.60 ± 221.23	145.53 ± 147.22	113.83 ± 99.96	84.78 ± 77.06	67.25 ± 67.64	55.02 ± 59.31	41.41 ± 52.56	34.91 ± 51.38	31.18 ± 48.63	27.92 ± 47.25	25.85 ± 44.77

*HU* Hounsfield units, mean value ± standard deviation.

**Figure 2:**
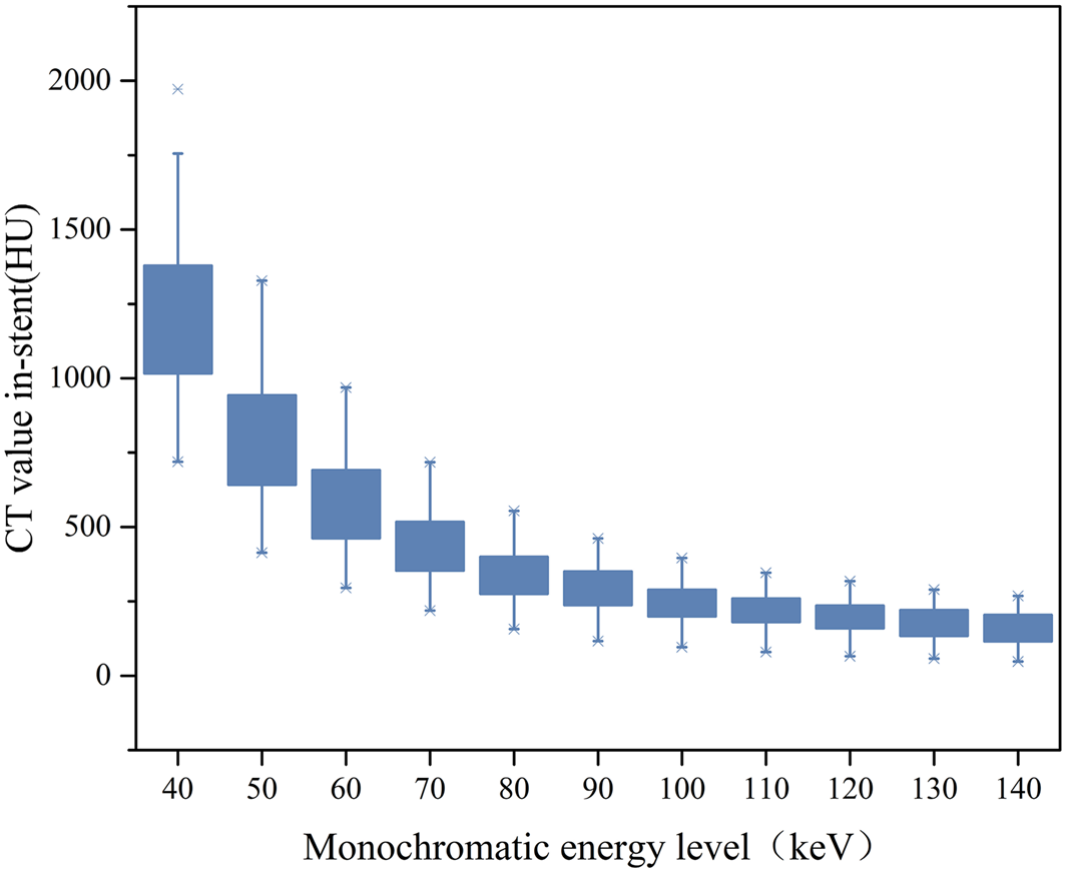
Comparison of CT value in-stent at monochromatic energy levels between 40 and 140 keV in 10-keV increments.

The mean CT value in-stent showed a decreasing trend with the increase in the monochromatic energy level, and at 80 keV energy level, the CT value was (345.54 ± 93.43) HU (Table 1, Fig. 2).

**Figure 3:**
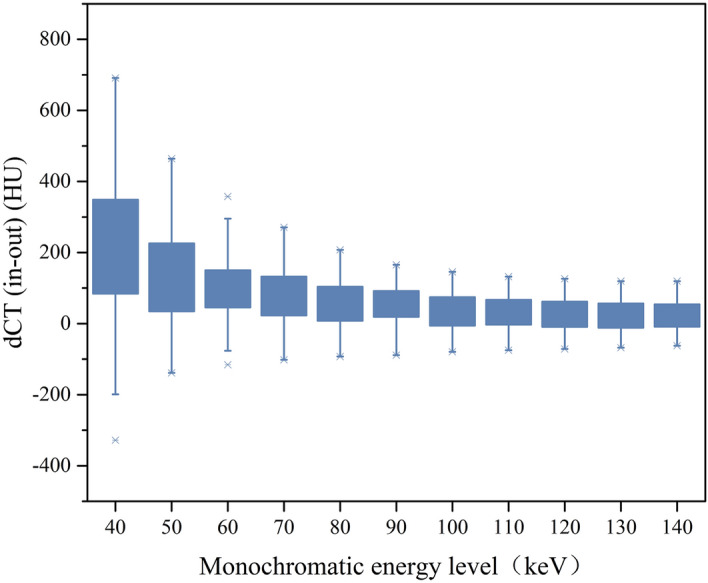
Comparison of dCT (in–out) at monochromatic energy levels between 40 and 140 keV in 10-keV increments.

At a higher monochromatic energy level, dCT (in–out) was smaller: 40–80 keV: (67.25 ± 67.64) HU–(174.60 ± 221.23) HU, 90–140 keV: (25.85 ± 44.77) HU–(55.02 ± 59.31) HU (Table 1, Fig. 3).

## Discussion

**Figure 4:**
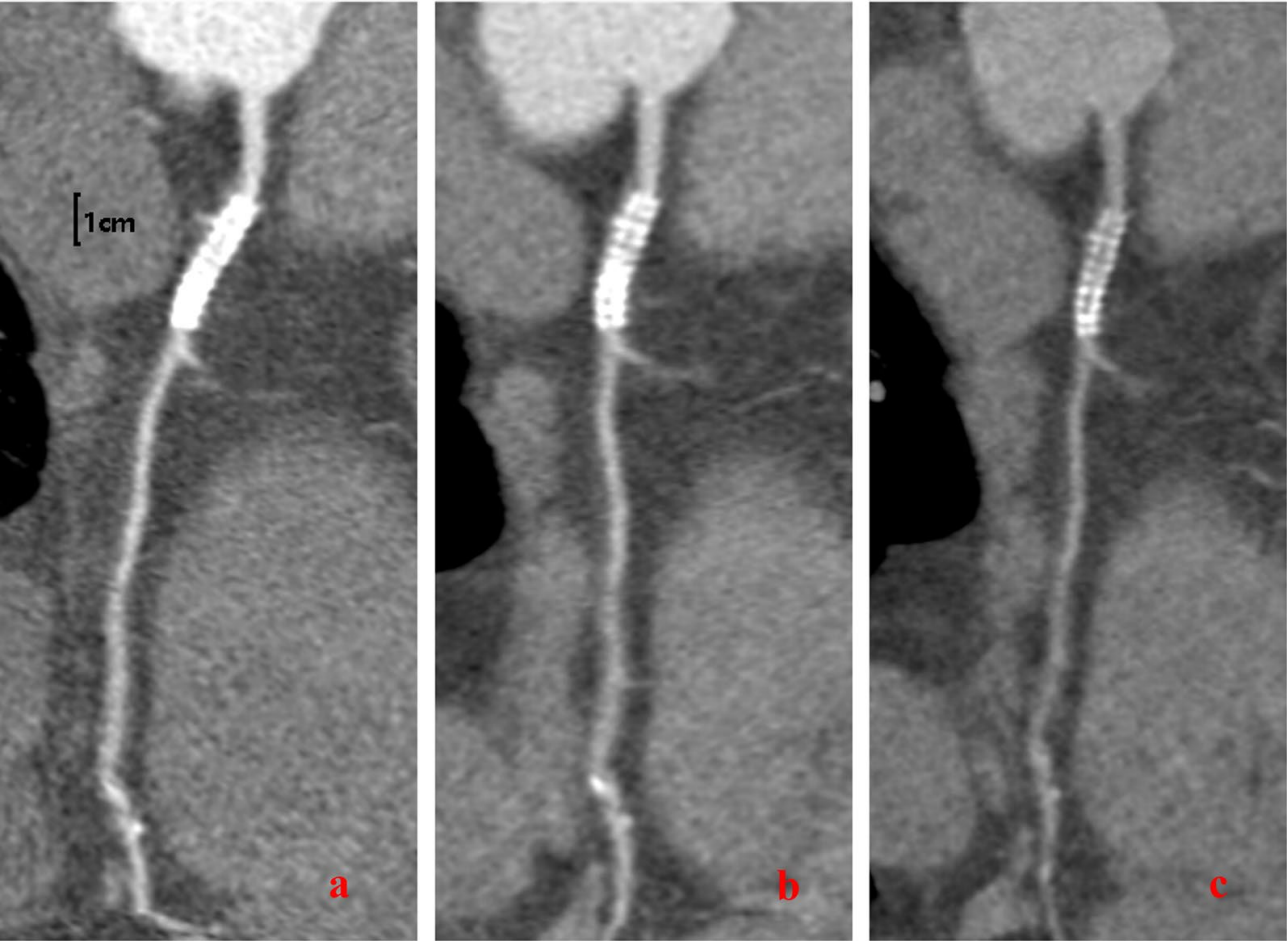
A 55-year-old man with a stent implanted in the right coronary artery (RCA). CPR of RCA: (**a**) virtual monoenergetic image (MEI) at a level of 40 keV, (**b**) at 80 keV, (**c**) at 120 keV. 40 keV MEIs were shown to have the hardening artifacts of the scaffold and wire harness, but the tissue was better. The hardening artifact of the wire harness of the 80 keV stent was reduced, and the coronary arteries showed good performance. The hardening artifact of the 120 keV stent wire harness was significantly inhibited, but the tissue contrast was weakened, and the coronary artery was less visible.

In the current study, we explored the optimal monochromatic energy level required in the dual-energy spectral CT for imaging coronary stents. The results showed that stent evaluation under dual-energy CT needed consider different monochromatic energy level together to improve the image quality and get the finest illustration. Our results indicated that with the increase in energy level, image noise, SNR and CNR of the in-stent lumen, and the CT value difference between the in-stent lumen and out-stent lumen dCT (in–out) were all decreased. At 80 keV, the mean CT value within the stent reached (345.24 ± 93.43) HU and dCT (in–out) started plateauing. In addition, the subjective image quality for the stents and vessels peaked at 80 keV (Fig. 4). Based on our results, we recommend routine generation of 80 keV monochromatic energy level reconstructions when performing dual-energy coronary computed tomography angiography (CCTA).

Stent thrombosis may occur early after PCI, and approximately 50% of ISR patients will not experience typical angina symptoms. It is extremely important to conduct effective follow-up, diagnosis and treatment within 1 year after PCI^12^. Due to its simple, noninvasive, and rapid characteristics, CCTA has become an important method and means of coronary artery disease assessment. Due to the influence of wire harness hardening artifacts and the inevitable partial volume effect, the CT value obtained by traditional mixed energy imaging has some errors to some extent. In the CCTA image, the metal artifact of the scaffold has the effect of magnifying the scaffold. Meanwhile, the inner diameter and area of the scaffold cavity decrease, and the CT value of the scaffold increases^13^.

A spectrum cardiac CT scan through the monophyletic instantaneous switching technology was used to obtain two groups of data at the same time, and allows the 101 groups of monochromatic energy images (40–140 keV) to choose any point in the virtual monochromatic reconstruction, can effectively reduce the beam hardening artifacts, making different tissue and lesions in the composition of the X-ray absorption differentially expressed and more apparent, can improve the image CNR at the same time, optimization, according to the different organizational structures to obtain a more stable and accurate CT value. The refinement of reconstruction techniques has greatly enhanced the potential of CT for coronary stent assessment, decreasing stent-related artefacts, and enhancing the delineation of the stent lumen. In addition to alleviating the harness hardening artifacts, Virtual monoenergetic imaging also allows radiologists to retrospectively select the energy that best compromises between increased vascular attenuation and acceptable image noise^14^.

Fan Y compared polychromatic images with monochromatic images in a study on dual-energy coronary CT imaging and found that the contrast and density resolution were higher at low monochromatic energy levels (50–65 keV), but the noise was also higher. In the meantime, the beam hardening artifacts at higher monochromatic energy levels (110–140 keV) showed higher suppression, but the density resolution decreased, and tissue contrast was weakened^15^. We Speculated that the monochromatic energy level reconstructions used in our study may provide such optimized visualization of both the stent and the lumen to improve the detection of ISR. In our study, SNR and CNR values of the in-stent lumen at a lower monochromatic energy level were higher than those of a higher monochromatic energy level. However, dCT (in–out) was lower at a higher monochromatic energy level, indicating a smaller artificial CT value increase due to the beam hardening artifacts caused by the stents. Secchi et al. found that the image quality score of the stent reached the highest at 120 keV (no artifacts and clearly displayed adjacent tissues) and the image noise reached the lowest at 80 keV on the second generation dual-source CT^16^. It was speculated that the best monochromatic energy reconstruction level for reducing stent artifacts was 80 keV after comparing among five energy levels (40–120 keV, 20 keV interval). In our study, more groups (11 groups) of monochromatic energy images were obtained, which might to be more extensive and credible than the above data collection. Normally, for vessel display in conventional CCTA, adequate enhancement in the vessel is essential for the detection of stenosis. Excessively low CT value will weaken the contrast between the blood vessel and surrounding tissue, and the coronary artery can be displayed clearly-when the CT value is approximately 350 HU. Our previous study also found that the adequate enhancement obtained at the lower monochromatic energy levels in the dual-energy spectral CT imaging (50 keV, 60 keV, 70 keV) was also important for a motion correction algorithm that requires the real-time tracking of coronary artery motion to be successful^17^. On the other hand, excessively high CT values in the vessel could also be detrimental. Soft plaques and calcified plaques could be difficult to be identified because of the lack of a clear display of these plaques in the case of the high intravascular CT value of the coronary arteries, which may lead to underestimation of the degree of stenosis of the coronary artery lumen^18^. In addition, the beam hardening artifacts were also more severe at lower photon energies. Our results suggested that the 80 keV monochromatic energy level was an optimal energy level to better balance the contrast enhancement and beam hardening artifacts suppression.

This study also has several limitations. First, the number of patients included in this study was small, which might lead to a bias and limits the effectiveness of the study to a certain extent. The sample size should be expanded. We are continuing to collect samples and hope to further our research and confirm them in larger patient populations. Second, this study included Cobalt–Chromium Alloy stents with a diameter of 3 mm, we did not evaluate the influencing factors such as different material and thickness and diameter of the stents; third, no diagnostic test comparing with the "gold standard" ICA was conducted, and the diagnostic efficacy could not be judged.

## Methods

### Clinical data

**Figure 5:**
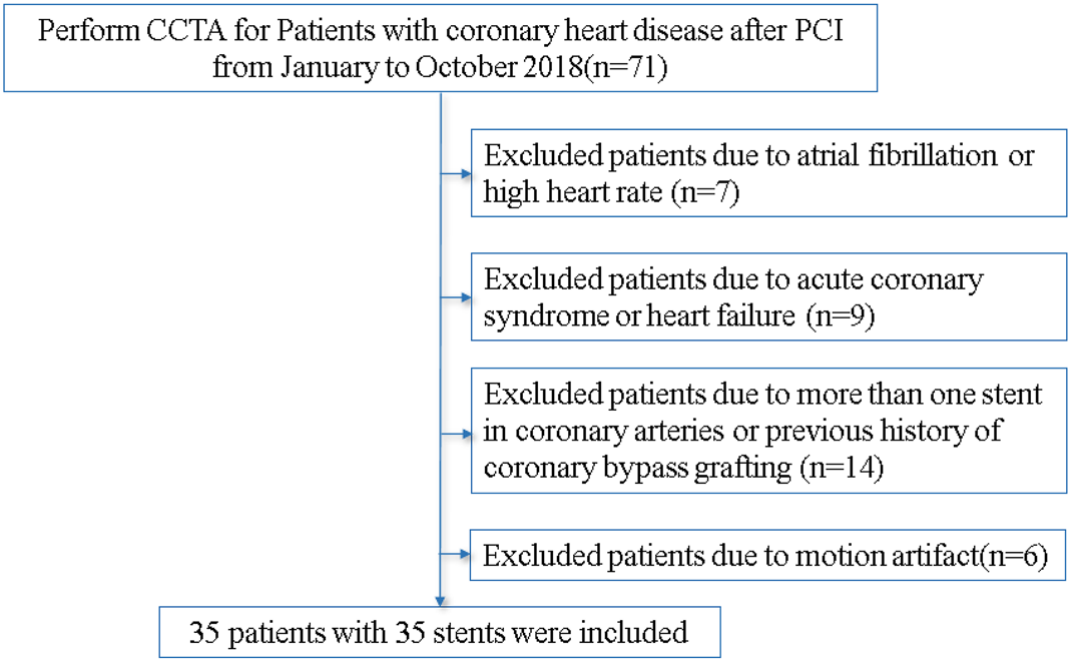
Flow diagram of patient recruitment.

This retrospective study was approved by the Ethics Committee of First Affiliated Hospital of Xinjiang Medical University. All methods were performed in accordance with the relevant guidelines and regulations and was performed in accordance with the Declaration of Helsinki. Informed consent was waived due to the characteristics of retrospective study’s design. The waiver was approved by the Ethics Committee of First Affiliated Hospital of Xinjiang Medical University. A total of 35 patients in our hospital were consecutively enrolled from January to October 2018. There were 31 males and 4 females, aged 42–68 years, with an average age of 57.3 ± 6.18 years. The inclusion criteria included (1) 3–6 months after PCI; (2) no cardiovascular adverse events occurred after PCI; (3) only one stent was implanted in each patient to avoid affecting the image analysis (such as overlapping stents or very small interval between stents); (4) Cobalt–Chromium Alloy stents with a diameter of 3 mm. The exclusion criteria were (1) arrhythmia (such as atrioventricular block and atrial fibrillation) or heart rate > 65 beats/min after oral metoprolol; (2) acute coronary syndrome (unstable angina pectoris or acute myocardial infarction) or cardiac dysfunction; (3) implantation of a pacemaker or internal defibrillator or previous history of coronary bypass grafting; (4) motion artifact of coronary arteries affecting stent evaluation. The study population is shown in Fig. 5.

### CT angiogram acquisition

All CCTA scans were performed on a 64 row CT scanner (GE Discovery 750 HDCT, GE Healthcare) capable of dual-energy spectral CT imaging mode. Patients' breath and heart rates were checked. Beta blockers (metoprolol p.o.) were administered before the scan for patients with heart rates above 65/min in the absence of contraindications. Patients were trained to hold their breath after a deep inhalation before the CCTA. The scan ranged from 2 cm below the tracheal carina to the level below the heart. The contrast injection protocol was to select different iodine delivery rates according to the patient body weight. The iodine delivery rate is the amount of comparator iodine injected per second (g I/s). The bolus tracking technique was used to trigger the contrast-enhanced CT scan with a region of interest (ROI) placed at the root of the aorta. The scan was triggered when the CT value in the ROI reached 100 HU and was started after 6 s of delay. The contrast-enhanced scanning used the dual-energy spectral CT imaging mode with a single-source, dual-tube voltage instantaneous switching between 80 and 140 kVp, tube current maximum limit 500 mA, gantry rotation speed 0.35 s, and the thickness and spacing of the layers were 0.625 mm, and range of the helical pitch was 0.16–0.22.

Eleven sets of monochromatic axial images with photon energies from 40 to 140 keV (with 10 keV interval) were reconstructed with a 40% adaptive statistical iterative reconstruction (40% ASIR), and CT angiograms were postprocessed on a three-dimensional image analysis workstation (GE Advantage Workstation 4.6, GE Healthcare). Datasets were evaluated with the transverse sections, multiplanar reformation (MPR), curved planar reformation (CPR), and maximum intensity projections (MIP) images.

### Image analysis

A randomized single blinded control was used for the image analysis. All images were independently interpreted by two radiologists who had been engaged in the interpretation of cardiovascular imaging for more than 5 years. A third senior radiologist with more than 10-year experience in cardiovascular radiology was asked to make the final decision in the case of any disagreement. The Likert 5-point scoring method was used to evaluate image quality using the MPR, CPR, and MIP images. The Likert scores were: 5-point, clear display of the stent structure and lumen, adequate enhancement with no beam hardening artifact, and excellent image quality; 4-point, slightly blurred stent edge and lumen display, adequate enhancement with slight beam hardening artifacts, and good image quality; 3-point, somewhat blurred stent edge and lumen display, adequate enhancement with some beam hardening artifacts, and fair image quality; 2-point, blurred stent edge and lumen display, no adequate enhancement, heavy beam hardening artifacts, poor image quality; 1-point, no clear separation between the stent and lumen, severe beam hardening artifacts, and non-diagnostic image quality. For the objective evaluation, the CT attenuation values of the aortic root and subcutaneous fat of the left chest wall, 5 points within the stent, at 5 mm from the proximal and distal ends of the stent in each monochromatic energy level were measured. The ROI for the aortic root was as large as possible, avoiding the calcification and non-calcified plaque area. Each stent was divided into four equal parts and select 5 points. The CT value of each point in the stent cavity was measured. The measurements were made on axial images of the stent and the ROI was circled manually to avoid the calcification. The average value of the 5-point in-stent lumen measurements and the 2-point out-stent measurements were used as the final value for the in-stent and out-stent CT attenuation value measurements, respectively. The CT value difference between the in-stent and out-stent lumen (dCT (in–out)), which reflects the artificial CT number increase due to the beam hardening effect caused by stents, was calculated by subtracting the out-stent lumen CT value from the in-stent lumen CT value. The SD in the subcutaneous fat of the left chest wall was defined as the image noise. SNR and CNR in-stent were calculated for each measurement as Eqs. (1) and (2):

1 $${\text{SNR}}_{{{\text{in-stent}}}} = \left( {{\text{HU}}_{{{\text{in-stent}}}} /{\text{SD}}_{{{\text{in-stent}}}} } \right)$$

2 $${\text{CNR}}_{{{\text{in-stent}}}} = \left( {{{\left( {{\text{HU}}_{{{\text{in-stent}}}} - {\text{HU}}_{{{\text{fat}}}} } \right)} \mathord{\left/ {\vphantom {{\left( {{\text{HU}}_{{{\text{in-stent}}}} - {\text{HU}}_{{{\text{fat}}}} } \right)} {{\text{SD}}_{{{\text{fat}}}} }}} \right. \kern-\nulldelimiterspace} {{\text{SD}}_{{{\text{fat}}}} }}} \right)$$

### Statistical analysis

Statistical analysis was performed by using SPSS 23.0 software (SPSS Inc, Chicago, IL, USA). Continuous variables were expressed as mean ± standard deviation, and categorical variables as counts. The reliability of the subjective assessment between two readers was evaluated by Chi-squared test. Repeated measures Analysis-of-variance (ANOVA) test was used to check differences of image quality scores, CT values in-stent, dCT (in–out), image noise, SNRs and CNRs among different monochromatic energy level. If getting significant results, least significant difference (LSD) was used. A two-tailed P-value < 0.05 is considered statistically significant.

## Conclusions

The 80 keV monochromatic images in the dual-energy spectral CT are optimal for imaging cardiac patients with stents after PCI, balancing the enhancement and SNR, CNR in the vessels while minimizing the beam hardening artifacts caused by the stents.

## Supplementary Information

Supplementary Information.
