# Supplementary material for: Exploring the best monochromatic energy level in dual energy spectral imaging for coronary stents after percutaneous coronary intervention
Source: Sci Rep. 2021 Sep 2;11:17576. doi: 10.1038/s41598-021-97035-7 (PMC8413443; doi:10.1038/s41598-021-97035-7)
Supplement: Supplementary file 1 — Supplementary Information. [file 41598_2021_97035_MOESM1_ESM.pdf]

Supplementary Materials

Exploring the best monochromatic energy level in dual energy spectral imaging for coronary stents after percutaneous coronary intervention

Qian Liu[1], Yajuan Wang[1,+], Haicheng Qi[1,+], Yaohui Yu[1,+], Yan Xing[1,*]

[1]Imaging center, the First Affiliated Hospital of Xinjiang Medical University, Urumqi, Xinjiang, China

Table S1 Comparison of image subjective assessment with different VMI reconstruction (Radiologist 1)

	1.	2.	3.	4.	5.	x^2	*P*
40 keV	0	0	10	21	4		
50 keV	0	0	9	22	4		
60 keV	0	0	8	22	5		
70 keV	0	0	4	24	7		
80 keV	0	0	2	25	8	29.888	0.072
90 keV	0	0	5	23	7		
100 keV	0	0	6	23	6		
110 keV	0	0	10	20	5		
120 keV	0	0	12	19	4		
130 keV	0	0	14	17	4		
140 keV	0	0	16	16	3		

Table S2 Comparison of image subjective assessment with different VMI reconstruction (Radiologist 1) ($\bar{x} \pm s$)

score	40 keV	50 keV	60 keV	70 keV	80 keV	90 keV	100 keV	110 keV	120 keV	130 keV	140 keV
$\bar{x}$	3.83	3.86	3.91	4.09	4.17	4.06	4.00	3.86	3.77	3.71	3.63
s	0.90	0.59	0.60	0.55	0.51	0.58	0.59	0.64	0.64	0.66	0.64
F						2.597					
P						0.005					

Supplementary Table S1 & S2 – Comparison of image subjective assessment with different VMI reconstruction (Radiologist 1). The subjective image quality for the stent and in-stent vessel was evaluated by two radiologists using a 5-point scale (5, excellent and 1, non-diagnostic).

Table S3 Comparison of image subjective assessment with different VMI reconstruction (Radiologist 2)

	1.	2.	3.	4.	5.	x^2	*P*
40 keV	0	0	9	22	4		
50 keV	0	0	9	21	5		
60 keV	0	0	8	21	6		
70 keV	0	0	6	23	6		
80 keV	0	0	3	24	8	26.129	0.162
90 keV	0	0	6	22	7		
100 keV	0	0	7	22	6		
110 keV	0	0	10	19	6		
120 keV	0	0	13	18	4		
130 keV	0	0	14	18	3		
140 keV	0	0	17	15	3		

Table S4 Comparison of image subjective assessment with different VMI reconstruction (Radiologist 2) ($\bar{x} \pm s$)

score	40 keV	50 keV	60 keV	70 keV	80 keV	90 keV	100 keV	110 keV	120 keV	130 keV	140 keV
$\bar{x}$	3.86	3.89	3.94	4.00	4.14	4.03	3.97	3.89	3.74	3.69	3.60
s	0.59	0.62	0.63	0.59	0.54	0.61	0.61	0.67	0.65	0.62	0.64
F						2.267					
P						0.014					

Supplementary Table S3 & S4 – Comparison of image subjective assessment with different VMI reconstruction (Radiologist 2). The subjective image quality for the stent and in-stent vessel was evaluated by two radiologists using a 5-point scale (5, excellent and 1, non-diagnostic).
